# Definitions and Standardization of a New Grading Scheme for Eyelid Contour Abnormalities after Trichiasis Surgery

**DOI:** 10.1371/journal.pntd.0001713

**Published:** 2012-06-26

**Authors:** Emily W. Gower, Sheila K. West, Sandra D. Cassard, Beatriz E. Munoz, Jennifer C. Harding, Shannath L. Merbs

**Affiliations:** 1 Departments of Epidemiology and Prevention and Ophthalmology, Wake Forest School of Medicine, Winston-Salem, North Carolina, United States of America; 2 Dana Center for Preventive Ophthalmology, Wilmer Eye Institute, Johns Hopkins University School of Medicine, Baltimore, Maryland, United States of America; 3 Division of Oculoplastics, Wilmer Eye Institute, Johns Hopkins University School of Medicine, Baltimore, Maryland, United States of America; Alfaisal University, Saudi Arabia

## Abstract

**Background:**

Clear definitions of outcomes following trichiasis surgery are critical for planning program evaluations and for identifying ways to improve trichiasis surgery. Eyelid contour abnormality is an important adverse outcome of surgery; however, no standard method has been described to categorize eyelid contour abnormalities.

**Methodology/Principal Findings:**

A classification system for eyelid contour abnormalities following surgery for trachomatous trichiasis was developed. To determine whether the grading was reproducible using the classification system, six-week postoperative photographs were reviewed by two senior graders to characterize severity of contour abnormalities. Sample photographs defining each contour abnormality category were compiled and used to train four new graders. All six graders independently graded a Standardization Set of 75 eyelids, which included a roughly equal distribution across the severity scale, and weighted kappa scores were calculated. Two hundred forty six-week postoperative photographs from an ongoing clinical trial were randomly selected for evaluating agreement across graders. Two months after initial grading, one grader regraded a subset of the 240 photographs to measure longer-term intra-observer agreement. The weighted kappa for agreement between the two senior graders was 0.80 (95% CI: 0.71–0.89). Among the Standardization Set, agreement between the senior graders and the 4 new graders showed weighted kappa scores ranging from 0.60–0.80. Among 240 eyes comprising the clinical trial dataset, agreement ranged from weighted kappa 0.70–0.71. Longer-term intra-observer agreement was weighted kappa 0.86 (95% CI: 0.80–0.92).

**Conclusions/Significance:**

The standard eyelid contour grading system we developed reproducibly delineates differing levels of contour abnormality. This grading system could be useful both for helping to evaluate trichiasis surgery outcomes in clinical trials and for evaluating trichiasis surgery programs.

## Author Summary

Approximately 8 million individuals worldwide suffer from trichiasis, a condition characterized by in-turned lashes that rub against the eye. Trichiasis is caused by repeated or prolonged ocular infection with *Chlamydia trachomatis*. Surgery is available to correct in-turned lashes. In most programmatic and research settings, the primary determinant of surgical success is whether or not lashes are touching the globe post-operatively. However, other surgical outcomes such as the contour of the eyelid are also important. Yet, no standard method for evaluating and reporting this outcome has been defined. In this study, we developed and tested a grading system for evaluating the severity of eyelid contour abnormalities after surgery using photographs of eyelids six weeks post-operatively. We found good agreement across photograph graders and also between field and photograph grades. This system should be useful in helping to standardize reporting of this outcome.

## Introduction

Trichiasis surgery is a critical component of the “SAFE” strategy, which is promoted by the World Health Organization (WHO) to eliminate blinding trachoma worldwide. Trachomatous trichiasis (TT) is a late-stage result of trachoma, a disease caused by ocular infection with *Chlamydia trachomatis*. Years of repeated infection cause the conjunctival lining of the eyelid to scar and contract, pulling the lashes inward and resulting in trichiasis. Eventually, trichiatic lashes may abrade the cornea, and when left uncorrected, can lead to irreversible blindness. Trichiasis can be corrected by any of several surgical approaches. One such approach, the bilamellar tarsal rotation (BLTR) procedure, is endorsed by the WHO [1], [2]. BLTR is the procedure of choice in many countries with trichiasis surgery programs that train non-physicians to perform trichiasis surgery.

Reported rates of unfavorable outcomes following surgery vary widely, with trichiasis recurrence rates ranging from 0–83% across individual surgeons in studies where patients were evaluated at least one year after surgery. Overall recurrence rates for these studies range from under 10% to over 50% [3]–[13]. Lagophthalmos after trichiasis surgery, also referred to as an “eyelid closure defect,” describes a condition where part of the globe is still visible when the eyelids are closed. Lagophthalmos may result from surgical over-correction, eyelid contour abnormality (ECA), pyogenic granuloma formation, or eyelid margin necrosis. Pyogenic granulomas are inflammatory masses on the tarsal conjunctiva that often develop as the body attempts to isolate a foreign body or irritant. Previous trials of trichiasis surgery have reported granuloma formation rates of 3–10% [8], [9], [14]. Eyelid margin necrosis, or accidental death of the eyelid margin tissue, is a known complication of transverse blepharotomy (full-thickness eyelid incision) and marginal rotation procedures. Necrosis and loss of the entire eyelid margin occur when the vascular source of the eyelid margin is violated [15]. Rates of lagophthalmos have not been reported consistently. In one of the first trials evaluating BLTR, 2 of 44 (5%) eyelids undergoing first-time BLTR surgery and 5 of 32 (16%) undergoing tarsal grooving, another procedure, had an eyelid closure defect 4 months after surgery [5]. Among patients who had prior trichiasis surgery, 29 of 68 eyes (38%) had an eyelid closure defect after repeat surgery. In a second trial by these researchers, excessive overcorrection resulted in an eyelid closure defect following 2% of tarsal rotation surgeries and 1% of tarsal advancement and rotation surgeries [4].

Of note, this second clinical trial provides the first published report of an additional finding that can be seen following trichiasis surgery but is not consistently reported. The manuscript describes “a poor cosmetic result from persistent unsightly ectropion” which occurred in 5% of eyelids following tarsal advancement and rotation and 2% of eyelids following tarsal rotation [4]. Another clinical trial describes “mild central notching without eyelid closure defect,” which occurred following approximately 6% of trichiasis surgeries (9 of 146 surgeries) [12]. Our team has noted similar findings while examining trichiasis patients post-operatively, both within the context of a clinical trial [14] and in follow up of surgeries performed in programmatic settings (data not published). Rates of these eyelid contour abnormalities seem to be substantially higher in the programmatic setting.

## Methods

### Objectives

While grading systems have been developed to describe trichiasis severity [16], [17], a systematic way of describing ECAs has not been published. The goal of this study was to develop a standardized, reproducible method for characterizing the severity of eyelid contour abnormalities.

### Ethics Statement

This study was approved by the Johns Hopkins and Wake Forest Schools of Medicine Institutional Review Boards and the National Institute for Medical Research (NIMR) in Tanzania. All participants provided written informed consent, and the study meets the tenets of the Declaration of Helsinki.

### Description of Data Source

This analysis relies on data from the Partnership for Rapid Elimination of Trachoma (PRET) Surgery trial. PRET is a randomized, masked clinical trial comparing outcomes following BLTR surgery using either standard instrumentation or the TT clamp [18]. Sutures were removed two weeks post-operatively. Six weeks, 12 months and 24 months after surgery, participants were examined for trichiasis recurrence, ECAs, and pyogenic granuloma formation. Field grades were recorded for each outcome at each visit. Photographs were taken pre-operatively, immediately post-operatively, and at each follow-up visit. For the current analysis, photographs from the six-week visit were utilized.

Photographs were taken in a standardized fashion. All follow up photographs were taken with the patient seated and the camera lens nine inches from the patient's superior orbital rim. Patients were asked to keep their head facing forward, but to look up as high as they could so that the upper eyelid margin could be seen. At the start of the PRET trial, one of the senior photograph graders (SLM) trained the field grader on classification of ECAs. At that time, we classified ECAs as mild or severe. However, during the course of developing the grading system, after the completion of the six-week period and the start of the one year follow-up period, we determined we could more finely delineate severity categories. In order to be able to directly compare the 6-week field and photograph grades, photographs graded as moderate or severe were combined into a single category called severe when comparing the field and photograph grades.

### Developing and Defining the Grading Scheme

Two senior graders, grader 1 (SLM) and grader 2 (EWG), developed a photograph grading scheme utilizing a subset of six-week post-operative photographs from the PRET trial. Clinical definitions characterizing the amount of vertical deviation and/or horizontal extent of the deviation were defined to delineate measurable differences in outcomes (Table 1). We found that also providing lay definitions assisted in training the photograph graders by providing a frame of reference. We asked them to imagine speaking to the patient at a conversational distance and then to determine whether they could see the ECA. Mild deviations would not be visible when standing at a conversational distance from the patient, while moderate would. Severe ECAs would be easily visible from across the room.

**Table 1:** Eyelid contour abnormality definitions.

Abnormality	Definition
**Mild**	Vertical deviation from the natural contour <1 mm in height (less than half the pupil height in daylight) and affecting <1/3 of horizontal eyelid length
**Moderate**	Vertical deviation from the natural contour 1–2 mm in height (about the pupil height in daylight) or affecting 1/3–2/3 of horizontal eyelid length
**Severe**	Vertical deviation from the natural contour >2 mm in height (more than the pupil height in daylight) or a defect >2/3 of the horizontal eyelid length

**Figure 1:**
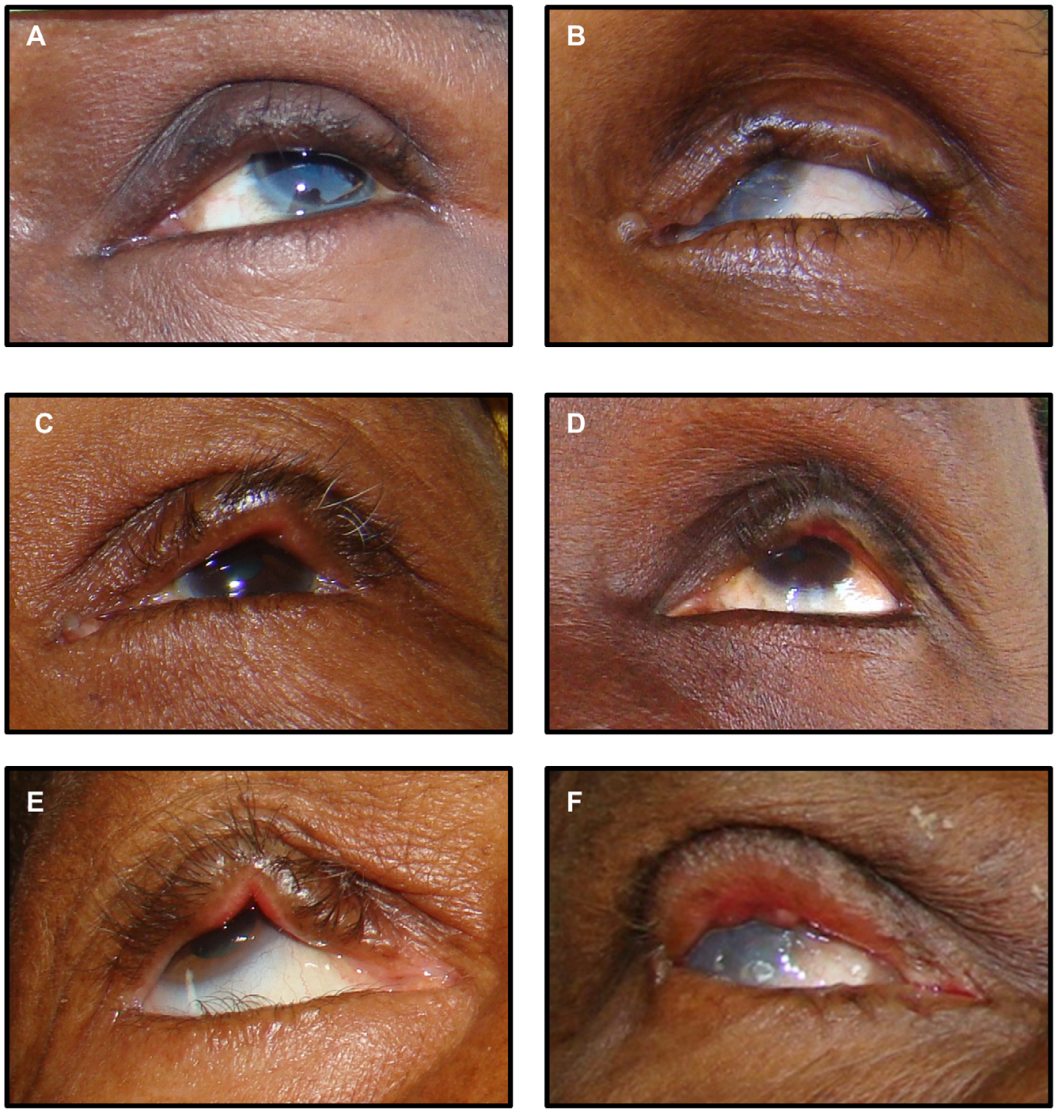
Standard photographs showing examples for each grading category. A and B) Mild eyelid contour abnormality C) Moderate abnormality based on height of deviation D) Moderate abnormality based on length of deviation. E) Severe abnormality based on height of deviation F) Severe abnormality based on length of deviation.

We compiled a set of standard photographs to illustrate abnormalities for each category. Photographs representing each category are provided in Figure 1. In addition, we created a Standardization Set of 75 eyelid photographs that were randomly selected with a roughly equal distribution across severity categories based on the clinical trial field grade. Agreement between graders 1 and 2 was measured for this 75-photograph standardization set. Graders 1 and 2 then met to adjudicate differences and to create gold standard grades for the Standardization Set. Several weeks later, grader 1 re-graded the Standardization Set.

### Training and Standardization

Next, we trained four additional graders (graders 3–6). Several practice grading sessions were conducted to familiarize graders with the variation in eyelid contour outcomes across patients. For each session, all graders independently graded a series of photos, and then the group met to review final grades for each eyelid and to discuss differences between graders. None of the photographs from the Standardization Set were utilized during training. Once graders 3–6 demonstrated a thorough understanding of the grading system, they independently graded the 75-photograph Standardization Set, and weighted kappas were calculated for each grader compared to the gold standard grade.

### Clinical Trial Photograph Grading

Following the standardization exercise, the six-week postoperative eyelid contours for each of the 3261 eyelids enrolled in the PRET trial with available six-week photographs were graded. Due to the large volume of photographs, we divided the work among 4 graders (graders 3–6), and each photograph was graded independently by two of the four graders. If the two grades agreed, this grade was designated the gold standard. If not, adjudication was performed by one of the senior graders (SLM), and her grade was designated the gold standard. Graders 4 and 6 graded the majority of the photographs. For the current analysis, 240 photographs (the Clinical Trial Subset) graded by both grader 4 and 6 were chosen randomly from the PRET 6-week photographs. We do not report findings for all eyelids in the PRET trial to avoid providing details on rates of outcomes, since the trial is ongoing. This Clinical Trial Subset was selected to exclude the 75 photographs in the Standardization Set and any photos utilized during training. In addition, photos selected for this Clinical Trial Subset were chosen to have a roughly equal distribution of ECA severities based on the gold standard grade. Weighted kappa scores for each grader compared to the gold standard grade were calculated for the Clinical Trial Subset. Two months following grading completion, grader 6 re-graded a random selection of 125 photographs from the PRET trial in order to measure longer-term repeatability of the grading system.

In order to compare field and photograph grades, moderate and severe contour abnormalities based on the gold standard photograph grades were combined into a group classified as severe. Field grades were compared to the gold standard photograph grades and weighted kappas were calculated. For all weighted kappa calculations, linear weights were used in order to be conservative, since linear weights penalize adjacent differences more than quadratic weights, resulting in lower kappa estimates, and because we believe the difference between each category is similar.

## Results

Agreement between the two senior graders who developed the grading system showed a weighted kappa of 0.80 (95% CI: 0.71–0.89) for the Standardization Set (Table 1), and intra-observer agreement for this set of photographs was 0.88 (95% CI: 0.81–0.95) for senior grader 1 (Table 2).

**Table 2:** Inter-observer agreement between two senior graders and intra-observer agreement for senior grader 1.

	Inter-observer Agreement				Intra-observer Agreement			
	Grader 2				2^nd^ Grade by Grader 1			
Grader 1	None	Mild	Moderate	Severe	None	Mild	Moderate	Severe
**None**	27	1	0	0	26	1	0	0
**Mild**	4	14	6	1	0	20	5	0
**Moderate**	0	1	8	4	0	1	10	1
**Severe**	0	0	0	9	0	0	0	9

Overall, agreement between graders 3–6 and the gold standard for the Standardization Set showed weighted kappa scores ranging from 0.60–0.79 (Table 3). In general, when the grader's score did not match the gold standard, the difference was off by one increment in severity. For example, grader 3 called three eyelids mild when the gold standard was moderate. Occasionally, the grader and the gold standard differed by two increments. Grader 3 had no instances where her grade was more than one increment different from the gold standard. Grader 4 had two instances where she graded severe and the gold standard was mild, three where she said moderate and the gold standard was normal, and one instance where she graded none and the gold standard was moderate. Graders 5 and 6 had two instances each where the difference was two increments, with one instance each where they graded more severely and one where they graded less severely than the gold standard. Graders 3, 4 and 6 tended to grade a bit more severely than the gold standard grade for the Standardization Set.

**Table 3:** Agreement between gold standard and each grader for eyelid contour abnormality photograph grading of the Standardization Set.

	Grader 3				Grader 4				Grader 5				Grader 6			
Gold Standard	None	Mild	Mod	Severe	None	Mild	Mod	Severe	None	Mild	Mod	Severe	None	Mild	Mod	Severe
**None**	25	3	0	0	25	1	2	0	22	5	1	0	19	8	1	0
**Mild**	2	17	6	0	6	11	6	2	10	8	7	0	1	19	5	0
**Mod**	0	3	7	3	0	0	5	8	1	3	2	7	1	2	8	2
**Severe**	0	0	1	8	0	0	0	9	0	0	0	9	0	0	1	8
**Weighted kappa**	0.79 (0.70, 0.88)				0.69 (0.59, 0.80)				0.60 (.48, .72)				0.72 (0.61, 0.84)			

Mod: Moderate.

For the Clinical Trial Subset, agreement between each grader and the gold standard grade ranged from 0.66–0.70 (Table 4). Longer-term intra-observer agreement for a subset of 125 of the clinical trial photographs was 0.86 (95% CI: 0.80–0.92) (Table 5).

**Table 4:** Agreement between the grader 4 and grader 6 and the gold standard grade for the clinical trial subset (240 photographs).

Gold Standard	Grader 4				Grader 6			
	None	Mild	Moderate	Severe	None	Mild	Moderate	Severe
**None**	52	11	5	1	55	14	13	1
**Mild**	5	32	12	3	5	40	17	6
**Moderate**	2	11	26	0	0	6	26	17
**Severe**	1	6	17	56	0	0	4	36

**Table 5:** Intra-observer agreement for 125 clinical trial photographs graded twice by grader 6, two months apart.

	Grade 2			
Grade 1	None	Mild	Moderate	Severe
**None**	38	2	0	0
**Mild**	7	31	2	0
**Moderate**	0	6	20	1
**Severe**	0	0	2	16

Weighted kappa: 0.86 (95%CI: 0.80–0.92).

Weighted kappa for the agreement between the gold standard photograph grade and the field grade was 0.68 (95% CI: 0.55–0.81) (Table 6). Differences were equally distributed between the field and photograph grade being more severe.

**Table 6:** Agreement between field and photograph grades for the Standardization Set.

	Photograph		
Field	None	Mild	Moderate/Severe
**None**	20	6	1
**Mild**	4	17	4
**Moderate/Severe**	0	4	16

Weighted kappa: 0.68 (0.55–0.81).

**Figure 2:**
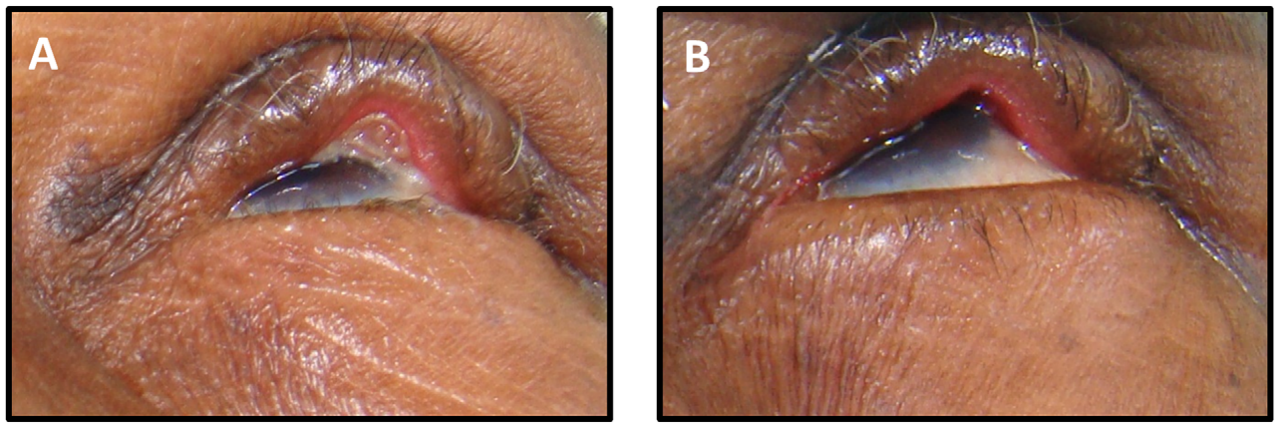
Photographs of an eyelid with an eyelid contour abnormality A) before and B) after granuloma excision.

Several eyelids had a granuloma at the six week visit. Pre- and post-excision photographs typically did not change the assigned grade (Figure 2).

## Discussion

The grading scheme described here provides a systematic, reproducible method for characterizing ECAs following trichiasis surgery. All weighted kappas were in the range of good to excellent, demonstrating that both good intra- and inter-observer agreement can be achieved with this straight-forward grading system based on photographs. The grading system allows for a central expert grader to evaluate photographs from a variety of areas without traveling to the field, thereby ensuring equal grading across areas. In addition, it allows for didactic classroom-based learning for training surgeons on how to evaluate ECA severity.

The 10 cases where the graders differed by 2 increments from the gold standard in the Standardization Set were examined for common characteristics. In three cases, there was a thickness to the eyelid that grader 4 apparently took into account when she graded the ECA 2 levels more severe than the gold standard. In four cases, the photograph was poor and difficult to grade.

Pyogenic granulomas were present in a number of eyelids with ECAs. In several instances, photographs were available for both before and after granuloma excision. In such cases, we instructed the graders to utilize the post-excision photographs. However, the severity of the ECA typically did not change based on which photograph was graded (Figure 2).

The photograph grades showed very good agreement with the field grades (weighted kappa, 0.68), suggesting that if the moderate and severe categories are collapsed, field grading of ECAs also could be done. Such agreement suggests that this modified grading system could be used in the field as part of the recommended monitoring program, and also could be used to supply feedback to new surgeons following training. In the current PRET trial, the full grading system (with moderate and severe differentiated) was implemented for the field grading at 12 and 24 months, and agreement of field grades between the field grader and grader 1 was excellent (kappa = 0.87; 95% CI: 0.76–0.98), suggesting that the full scale also can be utilized in the field. The level of agreement between field and photograph grades in this study (kappa = 0.68) is very similar to a previous study that evaluated the use of photographs to grade active trachoma (kappa = 0.71–0.74) [19]. Since that study was published, the use of photographs in grading active trachoma in clinical studies has become widespread.

This ECA grading system is a critical first step in providing trachoma control programs with a method for reporting on an important unfavorable surgical outcome. Few studies have reported eyelid closure defect rates [4], [5], [8], [9], and information on the prevalence and severity of ECAs is extremely limited. Providing a method for systematically reporting outcomes may help to increase reporting, and thereby improve understanding of the problems that can arise following trichiasis surgery.

Our study has some limitations. In some cases the photographic images did not capture the severity of the abnormality that might be better seen from photographs that included other angles. However, the correlation of the field and photograph grades suggested good agreement using the system as described here. Ideally we would have had one of the senior graders also grading the eyelids in the field to compare the two gold standards, but logistically that was not possible, as these data were collected over several months. In addition, our training and consensus building exercises were conducted over the period of two months. Had the graders continued to meet regularly to review grading, over time the inter-observer agreement may have increased. Finally, we did not use sophisticated digital image processing to measure the deviations precisely. It is possible that we could achieve greater precision if using such technology. However, this system was intended for use in low-technology settings, and as such, other areas of research may be better suited for determining whether agreement could be improved by image software processing.

ECAs are important for several reasons. First, in the severest form, ECAs can result in lagophthalmos, with the potential for corneal scarring and vision loss. Second, a poor aesthetic outcome of a friend or family member, such as a moderate or severe ECA, may be a key reason for the non-participation sometimes seen in trichiasis surgery programs [20]–[22], and yet this outcome has not been carefully studied. In trachoma-endemic areas, community links are typically quite strong and the primary mode of advertisement is by word of mouth. In many areas, surgical uptake remains very low, despite free surgical services being offered [21]–[23]. It is possible that one reason for low uptake is the appearance of the ECAs that other patients experience, since their neighbors typically will be aware that they have had surgery and will ask them about their experiences. To date, little is known about the effects of ECAs on long-term visual function and ocular health. Future research is needed to determine the effect of ECAs on vision and quality of life after trichiasis surgery.
